# Clinical Efficacy and Complications of Uterine Artery Embolization in Symptomatic Uterine Fibroids

**DOI:** 10.5539/gjhs.v8n7p245

**Published:** 2015-12-16

**Authors:** Mohammadgharib Salehi, Nasrin Jalilian, Ayoub Salehi, Mojgan Ayazi

**Affiliations:** 1 Department of Radiology, Imam Reza Hospital, Kermanshah University of Medical Sciences, Kermanshah, Iran; 2 Department of Gynecology and Obstetrics, Imam Reza Hospital, Kermanshah University of Medical Sciences, Kermanshah, Iran; 3 Kermanshah University of Medical Sciences, Kermanshah, Iran

**Keywords:** uterine artery embolization, uterine fibroma, complication

## Abstract

We decided to evaluate the efficacy and complications of uterine artery embolization (UAE) in patients with symptomatic uterine fibroids. Sixty-five premenopausal patients, without considering the fibroids size and its location, were treated by bilateral UAE. At baseline and after 3, 6, and 12 months MRI was obtained to determine the uterine length and fibroid diameter. In addition, symptoms of the patients were documented at these follow-up schedules. UAE was successful in 62 (95.4%) cases. Complete infarction rate of the fibroid was 83.1%. After 12 months, the uterine length showed a decrease of 55.7% (mean of 9.4 cm) and the diameter of the dominant fibroid revealed a decrease of 52.1% (mean of 3.4 cm). Menorrhagia improved in 45 cases (91.8%), abdominal mass in 24 cases (82.28%), urinary symptoms in 17 cases (85%), pelvic pain in 21 cases (84%), and dysmenorrhea in 25 cases (80.6%). At final follow-up performed after one year, complete infarction of the fibroma was demonstrated in 49 patients (83.1%). Two cases achieved successful pregnancy in the one year follow-up period. Five patients developed post-embolization syndrome which necessitated admission to the hospital. Twenty-two patients presented and complained of pain for which outpatient pain management was done. UAE was a successful treatment for uterine fibroids that preserved the uterus, had minimal complications, and required short hospitalization and recovery.

## 1. Introduction

Uterine leiomyomas (fibroids or myomas) are the most prevalent benign tumors in pelvic region in females (Baird et al., 2003). They are usually reported in pre-menopause women (Wise et al., 2004). They originate from the smooth muscle cells of the myometrium and result in symptoms such as abnormal uterine bleeding and pelvic pain or pressure (Tan, McClure, Tarnay, Johnson, Raman, 2014).

Treatment options available for uterine myomas include medical therapies such as gonadotropin-releasing hormone agonists (Minaguchi, Wong, Snabes, 2000) and surgical interventions including myomectomy. One of the relatively new interventional therapies available for treatment of such tumors is uterine artery ambolization (UAE). Previous studies reported that UAE is a safe minimally invasive method which resulted in 30-46% shrinkage in tumor size and alleviation of the symptoms (Gupta, Sinha, Lumsden, & Hickey, 2006). It has gained acceptance by many clinicians and studies regarding efficacy of this interventional method have been promising (Spies, 2013; Mara & Kubinova, 2014; Watkinson & Nicholson, 2007). Being a minimally invasive procedure and preserving the uterus are the two most common advantages of UAE implicated when compared to surgical procedures such as hysterectomy (Morris, 2008).

Despite advantages mentioned above, there is some controversy about application of UAE. In particular, some experts argue that UAE in fibroids with large size, for instance more than 10 cm, may be accompanied by complications such as infection, sepsis, and even death (Kim, M. Lee, M. S. Lee, Park, Wonq, Lee do, & K. H. Lee, 2012; Spies, Spector, Roth, Baker, Mauro, & Murphy-Skrynarz, 2002).

Therefore, we intended to study the clinical efficacy and complications of UAE amongst patients with symptomatic uterine fibroids. We hope that this report will be helpful for gynecologists and interventional radiologists who deal with such patients.

## 2. Materials and Methods

This study was done in collaboration between Obstetrics and Gynecology and Interventional Radiology Departments of our university hospital. A total of 65 patients who were diagnosed by applying the abdominal ultrasound performed by the attending gynecologist to be suffering from symptomatic uterine fibroids were consecutively selected. They were then referred to the Interventional Radiology services. Before UAE, magnetic resonance imaging (MRI) of the uterus was done to determine the fibroid size, its location, the uterine size and to exclude other concomitant conditions such as ovarian tumor. The baseline MRI revealed that the most common form of fibroma was of intramural (with or without submucosal or subserosal extension) type (80.6%), followed by submucosal fibroma (9 cases, 14.5%) and subserosal fibroma (3 cases, 4.8%).

UAE was done bilaterally using a Cobra 5.0-French catheter via accessing through the right femoral artery. After placing the catheter tip at the distal part of the uterine artery, the right and left arteries embolic agents were injected into the arteries (polyvinyl alcohol with or without gel foam) in 88.7%, Embo-Gold® (with or without gel foam) in 8.1%, and Embosphere® in 3.2% of the cases. One hour after injecting the embolic agent, the catheter was removed and the access site was packed for six hours.

The patients were discharged on the next day of admission. The patients were instructed to represent to the hospital in case of any complication development such as access site bleeding and the like. The patients were followed at 3, 6, and 12-month intervals after UAE using MRI in order to determine the uterine length and diameter of the fibroid. In case of more than 1 fibroid, the diameter of the dominant fibroid was considered in data collection. The total radiation dose was about 15 cGy which is equivalent to one CT scan or one barium enema.

The data were inserted to a checklist which in addition to information related to the uterine fibroids and the outcomes of the UAE, included demographic information of the patients and the related symptoms including menorrhagia, dysmenorrhea, abdominal mass sensation, and pelvic pain. The data were then inserted to the SPSS software for Windows (ver. 20.0). The descriptive indices such as frequency, percentage, mean and its standard deviation (SD) were used to express the data. The study protocol was approved by the Ethics Committee of our medical university. The patients firstly were informed about the study objectives as well as the procedure. Then, if agreed, written consent inform was obtained from them.

## 3. Results

Mean (±SD) age of the patients was 36.6 (±5.56) years (range, 21-47 years) and 28 were married and 11 had history of successful pregnancy. There was a past history of surgical myomectomy in 11 patients (17.4%). Twenty-two cases (34.9%) had used medical treatment, mostly Decapeptyl. Regarding the symptoms reported by the patients, menorrhagia was the most prevalent complaint (41 cases, 66.1%) followed by dysmenorrhea (8 cases, 12.9%), sensation of abdominal mass (7 cases, 11.3%), pelvic pain (3 cases, 4.8%), and increase in myoma size in one patient (1.6%). Also, 49 patients had severe menorrhagia, 29 had abdominal mass and distension, 20 had urinary system symptoms, 25 had pelvic pain, and 32 cases had dysmenorrhea. About 42.4% of the cases reported that they had problem in daily activities and required work leave due to fibroid-related symptoms.

Five patients developed post-embolization syndrome which necessitated admission to the hospital. Twenty-two patients presented and complained of pain for which outpatient pain management was done. Twenty-six cases reported vaginal necrotic secretions and infarcted fibroma was expelled via vagina in 4 cases. Amenorrhea was documented in 8 cases. In one patient who aged 44 and had family history of premature ovarian failure, amenorrhea was permanent. One patient who had submucosal myoma and UAE failure underwent abdominal myomectomy. Two cases had successful pregnancy during the follow-up period.

Uterine length of 6 cm in nulliparous women and 9 cm in multiparous women was considered as normal range. Baseline MRI revealed that mean uterine length was 121.2 mm (range: 9-20 cm). Mean diameter of dominant fibroid was 7.1 cm (range, 2.5-18 cm). Fifteen patients had single fibroid. MRI follow-up was achieved at 3, 6, and 12 months respectively among 88.1%, 78%, and 67.8% of the cases. After 3 months, mean uterine length showed a decrease of 33.4% (mean= 10.2 cm) and diameter of the dominant fibroid showed a decrease of 32.4% (mean of 4.8 cm). Symptoms of menorrhagia, abdominal mass, urinary system symptoms, pelvic pain, and dysmenorrhea improved in 33 (67.3%), 14 (48.3%), 14 (70%), 17 (68%), and 19 (59.4%) patients, respectively.

After 6 months, the uterine length showed a decrease of 41.2% (mean of 9.8 cm) and the diameter of the dominant fibroid revealed a decrease of 38% (mean of 4.4 cm). Menorrhagia improved in 42 cases (85.7%), abdominal mass in 20 cases (69%), urinary symptoms in 16 cases (80%), pelvic pain in 20 cases (80%), and dysmenorrhea in 23 cases (71.9%).

After 12 months, the uterine length showed a decrease of 55.7% (mean of 9.4 cm) and the diameter of the dominant fibroid revealed a decrease of 52.1% (mean of 3.4 cm). Menorrhagia improved in 45 cases (91.8%), abdominal mass in 24 cases (82.28%), urinary symptoms in 17 cases (85%), pelvic pain in 21 cases (84%), and dysmenorrhea in 25 cases (80.6%). At final follow-up performed after one year, complete infarction of the fibroma demonstrated in 49 patients (83.1%). In 2 cases (3.4%), complete enhancement of the fibroma was obvious which demonstrated UAE failure. In these 2 cases, the dominant fibroma was of subserosal type and in another patient was of submucosal type. In 2 cases (3.4%) with numerous myomas (more than 15) and dominant intramural fibroma with a diameter of more than 7 cm, increase in myoma growth was seen. In one patient, despite infarction in 60% of the dominant fibroma, the remainder of the fibroma continued to grow. In another patient, diameter decrease and complete infarction of the dominant fibroma was seen but half of the remainder fibromas continued to grow. Six patients showed partial response to UAE. In 2 cases with intramural dominant fibroma, some part of the fibroma showed enhancement but there was decrease in fibroma size. In 2 cases with several fibromas (more than 10 fibromas) and intramural fibroma, 60% of the fibromas showed infarction and the rest enhanced on MRI but fibroma growth increase was not seen. In 2 cases, decrease in uterine size and complete infarction of the fibroma was observed and only one non-dominant subserosal fibroma with a diameter of less than 4 cm did not show response to UAE.

**Figure 1:**
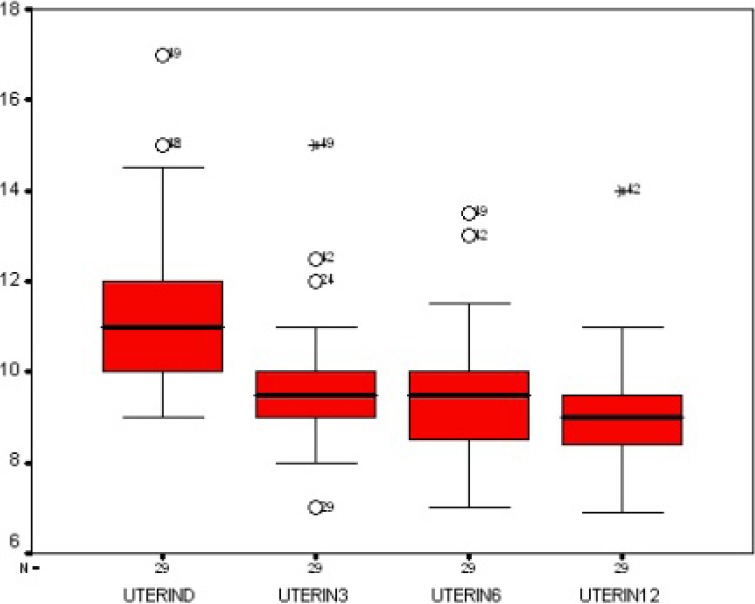
The changes in the uterine length at baseline and after 3, 6, and 12 months following uterine artery embolization

**Figure 2:**
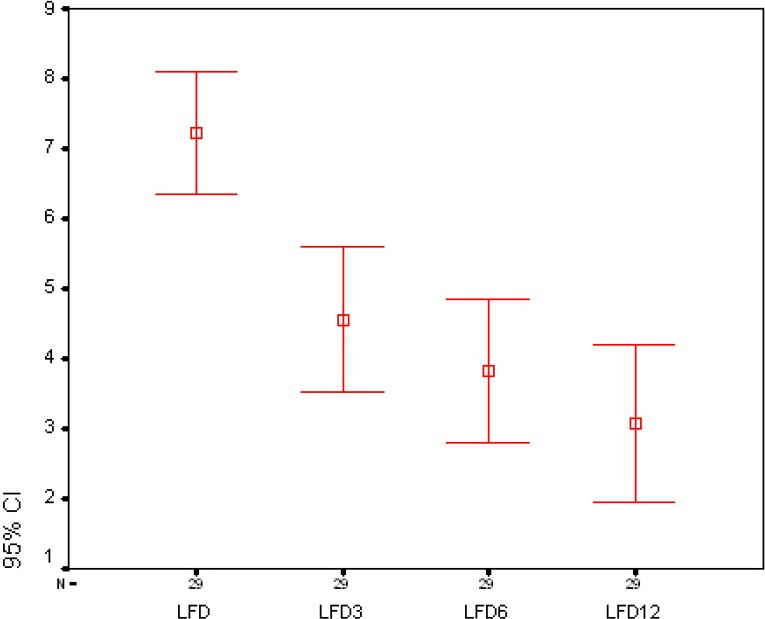
The changes in the dominant fibroid diameter at baseline and after 3, 6, and 12 months following uterine artery embolization

In Figures 1 and 2, the changes in the uterine length and fibroid diameter are respectively presented.

## 4. Discussion

According to the obtained findings, UAE was successful in about 95% of the patients. This figure is similar to a former report (Pron et al., 2003). They performed bilateral UAE and reported success rate of 97%. According to their experience, the most common cause for failure of bilateral embolization was variant anatomy. In another study (Mak et al., 2006) on 50 females showed successful rate of 98% (49/50 cases). Menorrhagia improved in 34 (84%) patients, dysmenorrhea in 28 (88%), pelvic pain in 18 (82%) and abdominal mass in 15 (83%). In our study, only one patient required surgical intervention for myomectomy. But in mentioned report (Mak et al., 2006), 8 patients (16%) underwent hysterectomies after uterine fibroid embolization

Similar to most studies, menorrhagia was the most common complaint reported by the patients. Here, we observed a very excellent result in improving this symptom. Also, overall uterine volume reduction has been reported in the range of 35% to 52% and fibroid volume reduction from 37% to 69% (Morris, 2008). These are compatible with the present findings. Some experts argue about desire for future pregnancy as a relative contraindication for UAE. We observed that 2 cases achieved successful pregnancy in the 1 year follow-up period. However, in a recent report on 66 women who desired a future pregnancy and were treated with UAE for symptomatic fibroids showed that just 1 out of 31 patients became pregnant (Torre et al., 2014). The authors advised that UAE is not appropriate method for younger patients with extensive fibroids who desire pregnancy in the future.

We observed 5 patients who developed post-embolization syndrome who required re-admission. The rate of this complication has been reported from 10-40%. This usually occurs in the first 2-week period after UAE and in some cases the pain lasts for more than 2 weeks (Morris, 2008).

The strength of this study relates to this fact that we included patients with different tumor sizes. Also we followed them for one year using MRI. However, we did not have a control group of surgical intervention such as hysterectomy to compare the results between the two groups. Also, it was not feasible to follow all patients at schedules follow-up visits as some of the patients did not present for follow-up. In conclusion, UAE was successful in managing the fibroids in symptomatic patients and preserved the uterus. No major peri-procedural complication occurred. We suggest this method as a safe and effective method to other interventional radiologists and gynecologists.
